# Early recognition of risk of critical adverse events based on deep neural decision gradient boosting

**DOI:** 10.3389/fpubh.2022.1065707

**Published:** 2023-01-26

**Authors:** Yu-wen Chen, Lin-quan Xu, Bin Yi

**Affiliations:** ^1^Chongqing Institute of Green and Intelligent Technology, Chinese Academy of Science, Chongqing, China; ^2^Department of Anesthesiology, Southwest Hospital, Third Military Medical University, Chongqing, China

**Keywords:** critical adverse events, deep neural network, multimodal information, XGBOOST, early recognition

## Abstract

**Introduction:**

Perioperative critical events will affect the quality of medical services and threaten the safety of patients. Using scientific methods to evaluate the perioperative risk of critical illness is of great significance for improving the quality of medical services and ensuring the safety of patients.

**Method:**

At present, the traditional scoring system is mainly used to predict the score of critical illness, which is mainly dependent on the judgment of doctors. The result is affected by doctors' knowledge and experience, and the accuracy is difficult to guarantee and has a serious lag. Besides, the statistical prediction method based on pure data type do not make use of the patient's diagnostic text information and cannot identify comprehensive risk factor. Therefore, this paper combines the text features extracted by deep neural network with the pure numerical type features extracted by XGBOOST to propose a deep neural decision gradient boosting model. Supervised learning was used to train the risk prediction model to analyze the occurrence of critical illness during the perioperative period for early warning.

**Results:**

We evaluated the proposed methods based on the real data of critical illness patients in one hospital from 2014 to 2018. The results showed that the critical disease risk prediction model based on multiple modes had faster convergence rate and better performance than the risk prediction model based on text data and pure data type.

**Discussion:**

Based on the machine learning method and multi-modal data of patients, this paper built a prediction model for critical adverse events in patients, so that the risk of critical events can be predicted for any patient directly based on the preoperative and intraoperative characteristic data. At present, this work only classifies and predicts the occurrence of critical illness during or after operation based on the preoperative examination data of patients, but does not discuss the specific time when the patient was critical illness, which is also the direction of our future work.

## 1. Introduction

The occurrence of critical illness in perioperative patients will not only increase the medical costs of patients, prolong the recovery time, and affect the rehabilitation results of patients (1, 2), but also even lead to the death of patients. At present, the incidence of various critical events during the perioperative period in China is as high as 12%, leading to the highest mortality rate of hospitalized patients at 1.1%. It is often difficult to timely predict critical events under the simple score warning system of critical events. This leads to the occurrence of critical events that are serious or late in the course of the disease, with greater treatment difficulty and limited intervention effect. Studies have shown that the occurrence of critical events within 30 days after surgery can reduce the median survival time of patients by 69% (3), and the long-term consequences of critical events have a great impact on patients' long-term survival and quality of life (4). A variety of critical illness events occurring during and after surgery are very common in clinical practice. Although some critical diseases cannot be avoided in view of the current level of medicine, their incidence rate can be significantly reduced if enough attention is given, and patients' suffering and death rate can be greatly reduced with the timely and correct treatment. Therefore, active risk prediction of critical events is helpful for early detection, early warning, diagnosis, and intervention, which is of great scientific significance and social value.

Currently, critical illness assessment in hospitals mainly adopts the scoring system, such as the trauma scoring system (5), Glasgow Prognostic Score System (6), acute respiratory distress syndrome score (ARDS Score) (7), disseminated intravascular coagulation score (DIC Score) (8), mortality prediction model (MPM) (9), acute physiology and chronic health evaluation II,III (APACHE II, III) (10), simplified acute physiology score (SAPS) (11), TISS score system (12), multiple organ dysfunction syndrome score (MODS) (13), and sequential organ failure assessment score (SOFA) (14). Although there are many clinical studies on critical illness scoring systems, their application in clinical practice is less. Because its accuracy mainly depends on the clinical experience of doctors or experts and the cognitive level of the disease, different experts or doctors often have a certain deviation in the diagnosis of the same disease for the same person. Besides, some critical care scoring systems (6, 13, 14) cannot obtain the required parameters in a short time, and the calculation is complex. Especially, the clinical information is far beyond the processing capacity of human brains because of its high rate of production and the large quantity of information, and the rapid change in the patient's condition.

Recently, artificial intelligence technology has been widely applied in the medical field (15–25). Statistical machine-learning methods based on sample data-driven models have also had widespread application in prediction models for critically ill patients (24, 26–28). It is a method that mines patient sample data through statistical learning methods to make a disease analysis based on the physical signs data after the processing and transformation of the data, so as to predict critical illness. Currently, severe risk prediction based on machine learning is divided into evaluating with structured text data and evaluating with unstructured text data. The study by Krittanawong et al. (29) used structured data for risk assessment of critical illness. The patient's disease characteristics were obtained based on the disease feature extraction method designed by professionals on the data set, and the patients were stratified according to the risk level of the disease by using the machine learning method. This method has been widely studied and applied in clinical practice. The study by Schnabel et al. (30) used the method of big data analysis to evaluate diseases from learning features in a large number of structured data sets. Although structured data facilitate the development of health monitoring applications with data mining methods, unstructured text data records important information about patients' diseases. The study by Yang and Garibaldi (31) mined features of coronary artery disease from clinical record data using a text-mining method to predict the risk of coronary artery disease. The study by Evans et al. (32) used a natural language processing method to identify risk features of cardiovascular disease. With the development of computer technology, studies of AI are no longer limited to basic algorithms, but have begun to upgrade and integrate algorithms (33–35). Upgraded algorithms have been applied in the fields of motion planning (36), the Internet (37), and medicine (38).

The concept of information fusion appeared in the 1920s, and after 60 years of development, it finally became a special technology in the 1980s. Information fusion is an inherent feature of an organism. It is the basis for an organism to perceive its environment and respond to it, as well as the basic ability of an organism to evolve and survive. The general definition of information fusion is a process through which data obtained by multiple sensors (including soft sensors) in chronological order are automatically analyzed and optimized according to certain criteria, and finally, the information for decision-making and estimation is obtained by using computer technology. Disease risk assessment based on a simple text data drive or numerical type drive has not fully integrated the preoperative and intraoperative relevant data of patients and cannot fully reflect the actual condition of patients. Using information fusion technology to analyze, process, and fuse medical data, and to analyze disease types and diseases, can effectively improve the accuracy of disease prediction. Therefore, this article adopts the machine learning method to model the risk prediction of critical illness occurrence before and during operation for patients based on the relevant diagnosis and treatment data of patients, so as to build an extensible, low-cost, and effective machine-learning critical illness event prediction solution. This article is not intended to advocate that artificial intelligence will replace the work of doctors, but merely to show how artificial intelligence and machine learning can help humans to predict various critical diseases early and minimize manual operation with a low-cost, efficient, and accurate method.

In this article: (1) we propose a deep neural decision gradient boosting model to predict critical illness; (2) we evaluated the proposed methods based on the real data of critical illness; (3) critical illness; and (4) the experimental results show that the disease risk prediction model based on multimodality has faster convergence speed and better performance than the risk prediction model based on text data and pure data type.

The remainder of this article is organized as follows. First, we elaborate on the method proposed in this article. Next, this article demonstrates the effectiveness of the method proposed in this article from the experimental results. Finally, we conclude and outline future work.

## 2. Methods

### 2.1. Model structure

**Figure 1:**
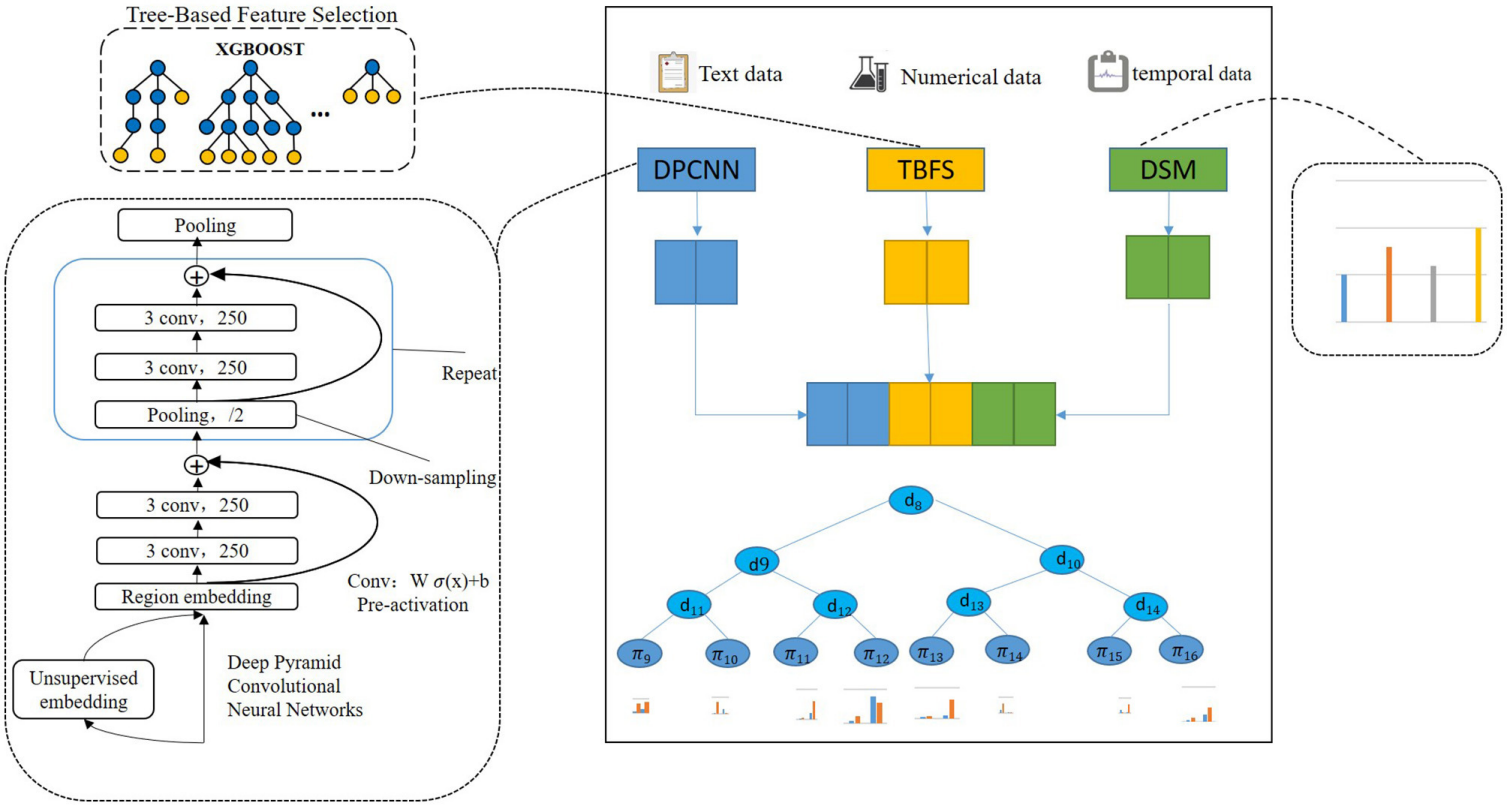
Critical illness risk prediction based on deep neural decision gradient boosting.

In this study, text data and structured data were used to predict the risk of critical illness, increasing the stability of the model. The multimodal disease risk assessment model uses unstructured text data and structured historical data from patients' medical data to analyze whether patients will suffer from critical illness. The risk assessment model is trained by using supervised learning, with input data represented as X, both structured and unstructured. Particularly, structured data includes basic information about the patient (e.g., age, gender), preoperative examination data, and intraoperative monitoring sequence data. Unstructured medical text data mainly includes the chief complaint of the patient, preoperative-clinical diagnosis, and conclusions from the patient examination (e.g., echocardiography-examination, electrocardiogram-examination, chest X-ray-examination). The disease risk assessment model of multi-modal medical data needs to extract unstructured text data features and structured data features, and integrate the two types of features. In this article, a multimodal disease risk assessment model based on deep neural networks and XGBOOST is proposed to extract text features from unstructured text data by using a deep neural network and data-driven feature learning. The pure data type features are extracted by using the method of feature engineering. Specifically, the feature fusion layer is designed in the predictive model to determine the connection parameters between structured data features and unstructured data features and classifiers through supervised learning. Figure 1 is the structure diagram of critical illness risk prediction based on multi-mode data fusion. From top to bottom, it shows the input layer of patient data, feature learning layer, feature fusion layer, and classification output layer.

The left branch of the input layer and the feature learning layer is unstructured text data feature learning. A deep neural network is used to learn the features of Chinese text data. In the middle is the learning of numerical type characteristics related to the preoperative test of patients and population information. The XGBOOST algorithm is used to select the medical data of pure data type and regulate the input data. We call this tree-based feature selection (TBFS). The sequence data and feature learning of intraoperative monitoring of patients are on the right. The method of feature statistics and abnormal time statistics are used to extract features of intraoperative monitoring attributes based on the data statistical method (DSM). The data fusion layer fuses the learned text features and structured data features, while the full connection layer fuses the features. The input data are the output of the deep neural network layer, the output of XGBOOST feature selection, and the intraoperative statistical feature quantity, while the output data are multi-mode data features. The XGBOOST classifier is used as the classification output layer, with the input being the multimodal data characteristics and the output being disease risk assessment results.

### 2.2. Feature extraction

To predict the risk of critical illness based on the diagnosis and treatment of patients, whether the data is structured data, unstructured data, or time series data, data features need to be extracted, that is, features need to be represented in the original data. Feature representation can be divided into manual design features and data-driven feature learning. The method of manual features is to extract the features from the original data or design the features based on the experience accumulated by a human when completing the task. The learning method of data-driven features uses a large amount of data to train the feature learning algorithm and finally obtain the representation method of the features in the original data. In this article, the preoperative text features and numerical features of patients were extracted in a data-driven way, and the intraoperative monitoring data features were extracted in the way of feature engineering combined with doctors' experience, described in the following paragraphs.

#### 2.2.1. Preoperative text feature extraction

**Figure 2:**
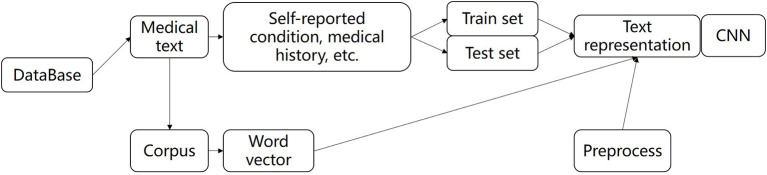
The flow of text feature extraction.

Analyzing the characteristics of medical text data plays an important role in predicting the risk of critical illness. In this article, we used the method of the word vector (39, 40) and deep learning model to learn the text data features. The method of establishing critical text characteristics based on the convolution neural network learning model is applied to general critical risk prediction. A text feature learning model based on a convolutional neural network is built and applied to the risk prediction of universal critical illness. As a data-driven feature representation learning method, deep learning combined with word vectors can effectively obtain the context information for text data and obtain the representation of text features, which has great advantages in text classification, text emotion analysis, and other natural language processing tasks. Collobert proposed a general deep learning structure for natural language analysis (41). Lai proposed a model of recurrent neural network (RNN), which used word vectors to represent input data and combined with the advantages of recurrent neural network and Convolutional neural network (CNN), it performs well in tasks of text classification, text emotion classification (42). Santos used character vectors and trained word vectors to design a deep convolutional neural network for the classification of emotion in a short text. The above research shows that the combination of word vectors and deep learning methods has great advantages in text analysis (43). The text data analysis of the diagnosis of the patient needs to be normalized. The text diagnosis data related to all patients were cleaned and preprocessed. The processed data were used as a corpus and trained by word2vec to obtain word vectors, as shown in Figure 2.

The preoperative clinical text data of all patients were digitally represented with word vectors by preprocessing methods, which were divided into the training set and test set, and input depth neural network for training and testing, and finally extracted text features. In this paper, we used deep pyramid convolutional neural networks (DPCNN) to extract the features. DPCNN (44) is a low-complexity word-level deep convolutional neural network architecture for text categorization that can efficiently represent long-range associations in text. The specific structure is shown in Figure 1. The first layer performs text region embedding, which generalizes commonly used word embedding to the embedding of text regions covering one or more words. It is followed by stacking of convolution blocks (two convolution layers and a shortcut) interleaved with pooling layers with stride 2 for down sampling. The final pooling layer aggregates internal data for each document into one vector. It uses max pooling for all pooling layers. DPCNN can effectively extract the features of long-distance relationship in the text, with low complexity and to better effect than the previous CNN structure.

#### 2.2.2. Preoperative data type attribute reduction feature extraction

**Figure 3:**
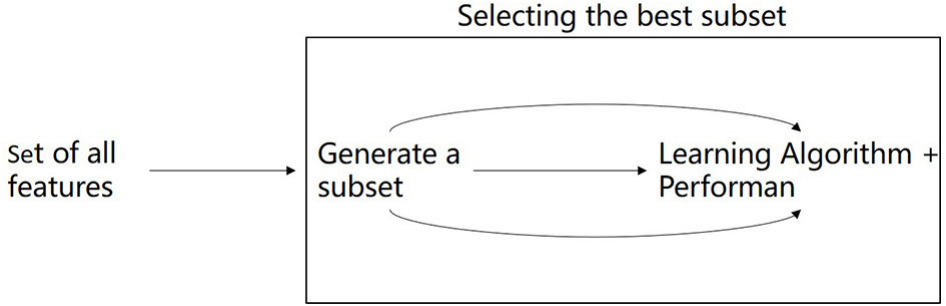
Selecting the best feature.

There are many preoperative test attributes for patients. The greater the number of attributes in the critical illness diagnosis using the machine learning method, the more complicated the construction of the prediction model will be, and the more time will be spent in the subsequent model training. Besides, since the attributes of critical illness are interrelated and mutually restricted, it is crucial to screen out the attributes with the most information. Therefore, in the construction of the critical illness prediction model, the attribute reduction of data in the dataset should be carried out to reduce the dimension of the attribute and the complexity of the data. Attribute reduction selects the most representative part of attribute features from the feature set of original collected patient data through certain methods. As the number of attribute features increases, the accuracy of the final result will be affected. Therefore, attribute reduction needs to filter out the redundant feature information to improve the accuracy of the target result on the premise that the data is true. In this article, the XGBOOST algorithm is used to select the feature importance of the preoperative structural test data as shown in Figure 3.

#### 2.2.3. Feature extraction of intraoperative monitoring data

The role of structured data from intraoperative monitoring in the risk assessment of critical illness cannot be ignored. Therefore, this article converts intraoperative monitoring data into corresponding fixed features through the method of feature engineering. The methods used in the model include statistical outliers and statistical characteristics of patient monitoring attributes. Specifically, the outliers are the amount of time that the patient's monitoring attribute data is out of the normal range (measured in minutes), the normal range is shown in Table 1, and the statistical characteristics include the maximum, minimum, mean, variance, standard deviation, kurtosis, and skewness of each attribute.


$$\begin{array}{l} \;\mu =\frac{1}{T}\sum_{i=\;1}^{T}x_{i}.\\ \;\sigma^{2}=\sum_{i=1}^{T}\frac{1}{T}{\left(x_{i}-\mu \right)}^{2}.\\ Skewness\left(X\right)=E\left[{\left(\frac{X-\mu }{\sigma }\right)}^{3}\right]=\frac{1}{T}\sum_{i=1}^{T}\frac{{\left(x_{i}-\mu \right)}^{3}}{\sigma^{3}}.\\ Kurtosis\left(X\right)=E\left[{\left(\frac{X-\mu }{\sigma }\right)}^{4}\right]=\frac{1}{T}\sum_{i=1}^{T}\frac{{\left(x_{i}-\mu \right)}^{4}}{\sigma^{4}}. \end{array}$$

**Table 1:** Normal value range of patient monitoring attributes during operation.

**Number**	**Attribute name**	**Value interval**
1	Heart rate (HR)	[50, 100]
2	Systolic blood pressure (SBP)	[90, 140]
3	Diastolic blood pressure (DBP)	[60, 90]
4	Central venous pressure (CVP)	[5, 12]
5	Respiratory rate (RR)	[12, 20]
6	ETCO_2_	[35, 45]
7	Temperature (T)	[36.2, 37.2]
8	Rectal temperature (RT)	[36.5, 37.7]
9	SpO_2_	[95, 100]
10	Ambulatory diastolic blood pressure (ADBP)	[60, 90]
11	PULSE	[60, 100]
12	Mean arterial pressure (MAP)	[60, 10,000]
13	Mean blood pressure (MBP)	[60, 10,000]
14	Arterial systolic blood pressure (ASBP)	[90, 140]
15	Systolic pressure of pulmonary artery (SPPA)	[15, 30]

### 2.3. Classification of the model

XGBOOST (45) is used to predict critical illness, which is an integrated learning method proposed by Chen based on GBDT (46). The improvement of the XGBOOST algorithm to the GBDT algorithm is that the second derivative is used to calculate the objective function in the process of model optimization. Furthermore, the regularization term is added to the objective function to prevent the algorithm from over-fitting in the training process. Moreover, the XGBOOST algorithm uses the idea of a random forest for reference in the training process and does not use all samples in the iteration process or every iteration. The generalization ability of the model is effectively improved by sampling all the features of the samples and training some of the features of the samples. Different patients have different test and intraoperative monitoring data and if the inspection attribute of each patient's data is missing, XGBOOST can solve the model of missing value. As such, this article chose XGBOOST as the classifier of the model as shown in Figure 1.

## 3. Experiment

### 3.1. Data description and preprocessing

The data for this experiment were from real critical patients in a hospital from 2014 to 2018, including 609 patients with heart failure, 171 patients with hepatic failure, 253 patients with kidney failure, 194 patients with respiratory failure, and 4,223 patients in the control group. Each patient sample contained ~200 descriptive indicators including age, gender, examination, and surgical information. There are different indicators in different critical cases. Additionally, for the electronic patient records recorded in the preoperative and postoperative repeated inspection and examination data, we only selected the valid data from the last preoperative test and examination of the patient to construct the dataset. If the data item from the test or examination closest to the operation was null, the test and examination values within 2 weeks were extracted in chronological order for filling. If the patient did not perform a test or examination index, the value is filled. For example, if the surgical date of a patient is 28/10/2019, the results of the most recent (26/10/2019) test and examination before 28/10/2019 are extracted. If the test and examination value is null, the values of the test and examination within 2 weeks are extracted in chronological order for filling. Numerical test data were normalized, and all index attribute values were converted to the interval (0, 1). For the textual check conclusion data, in this article, the textual data was cleaned and the character symbol was removed. Since the indexes of intraoperative monitoring for different critical cases are different, this article first calculates the indexes of intraoperative monitoring for such critical cases through statistical methods. The largest number of the first n indicators were selected as analysis indicators (the number of n was determined according to different critical cases, see the Section 3).

### 3.2. Experiments and results

We used three datasets, i.e., the structured data set, the unstructured data set, and the multi-modal data set. The experiment was divided into four groups, as shown in Table 2.

**Table 2:** Group experiment description.

**Group name**	**Description**
G1	Structured dataset, including preoperative patient testing and demographic information
G2	Temporal dataset and intraoperative monitoring temporal dataset
G3	Unstructured text data
G4	G1, G2, and G3 fusion datasets, including structured, unstructured, and monitored temporal datasets

G1: Structured dataset. An algorithm based on the tree model was used to extract the feature attributes, and the machine learning classifier was used to judge whether the patient was in critical condition.

G2: Temporal dataset. The abnormal time and monitoring statistics were extracted based on the feature engineering, and the prediction was made based on the machine learning classifier.

When extracting features of unstructured data from the G3 and G4 datasets, word vector representation was obtained through training, and feature representation of text was learned through a convolutional neural network.

In this article, the cross-validation method was used to train the model. Patient data were divided into training (70%), validation (20%), and test data set (10%). Furthermore, we compared the logistic regression, Gaussian Naïve Bayes, k-nearest neighbor, random forest, and Adaboost classifier values, commonly used in the medical field, in the experiment.

#### 3.2.1. Experiment 1: Structured data experiments and results

**Figure 4:**
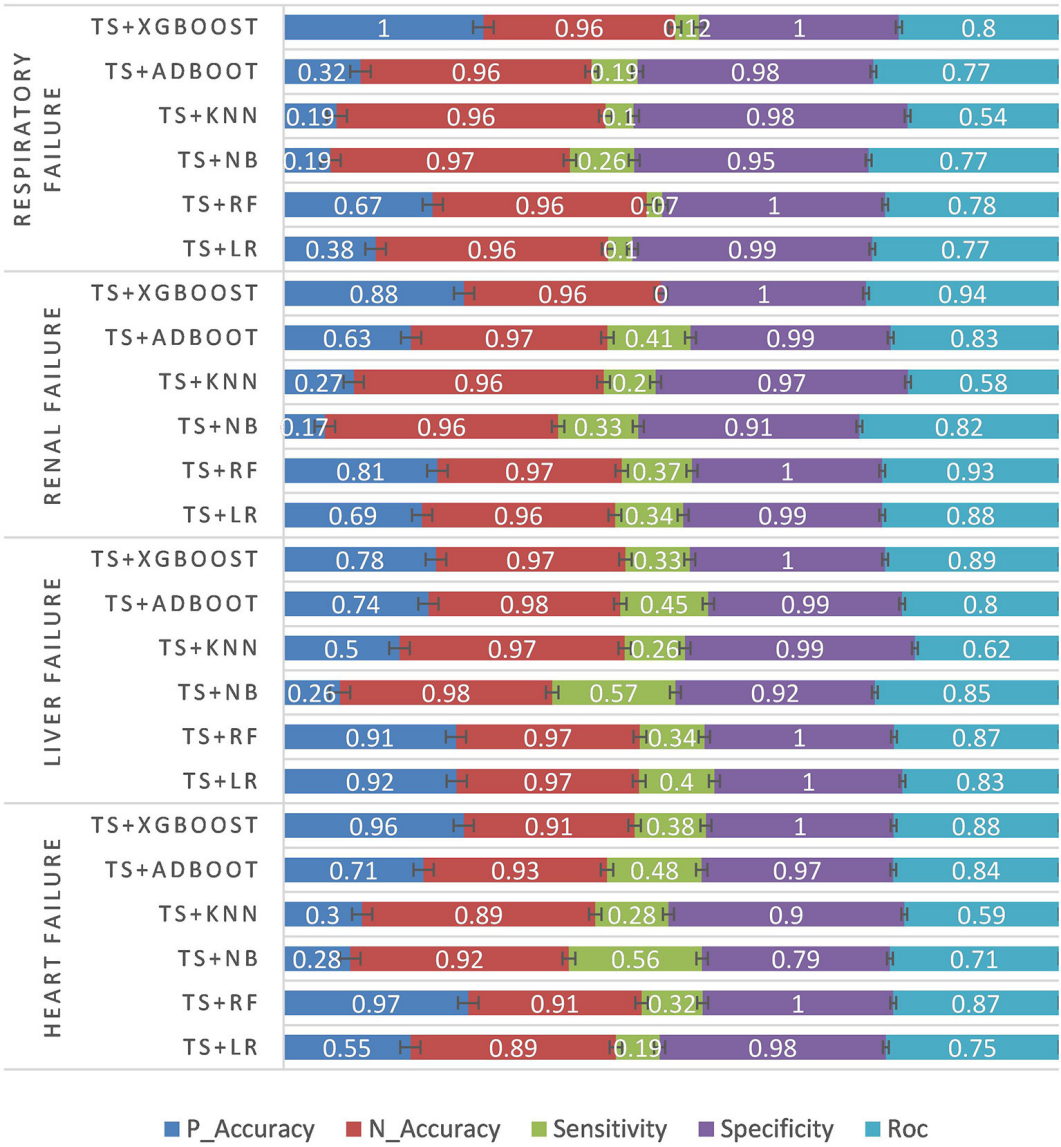
Structured data experiment results.

The first experiment was conducted based on the preoperative structured data of patients. The experimental results are as follows. P_Accuracy is the abbreviation of positive accuracy, N_Accuracy is the abbreviation of Negative accuracy, and TS is the abbreviation of TBFS. The experimental results are shown in Table 3 and Figure 4.

**Table 3:** Structured data experiments results.

**Illness**	**Classifier**	**P_Accuracy**	**N_Accuracy**	**Sensitivity**	**Specificity**	**ROC**
Heart failure	TS + LR	0.55	0.89	0.19	0.98	0.75
	TS + RF	0.97	0.91	0.32	1	0.87
	TS + NB	0.28	0.92	0.56	0.79	0.71
	TS + KNN	0.3	0.89	0.28	0.9	0.59
	TS + Adaboost	0.71	0.93	0.48	0.97	0.84
	TS + XGBOOST	0.96	0.91	0.38	1	0.88
Liver failure	TS + LR	0.92	0.97	0.4	1	0.83
	TS + RF	0.91	0.97	0.34	1	0.87
	TS + NB	0.26	0.98	0.57	0.92	0.85
	TS + KNN	0.5	0.97	0.26	0.99	0.62
	TS + Adaboost	0.74	0.98	0.45	0.99	0.8
	TS + XGBOOST	0.78	0.97	0.33	1	0.89
Renal failure	TS + LR	0.69	0.96	0.34	0.99	0.88
	TS + RF	0.81	0.97	0.37	1	0.93
	TS + NB	0.17	0.96	0.33	0.91	0.82
	TS + KNN	0.27	0.96	0.2	0.97	0.58
	TS + Adaboost	0.63	0.97	0.41	0.99	0.83
	TS + XGBOOST	0.88	0.96	0.3.	1	0.94
Respiratory failure	TS + LR	0.38	0.96	0.1	0.99	0.77
	TS + RF	0.67	0.96	0.07	1	0.78
	TS + NB	0.19	0.97	0.26	0.95	0.77
	TS + KNN	0.19	0.96	0.1	0.98	0.54
	TS + Adaboost	0.32	0.96	0.19	0.98	0.77
	TS + XGBOOST	1	0.96	0.12	1	0.8

#### 3.2.2. Experiment 2: Temporal data experiments and results

**Figure 5:**
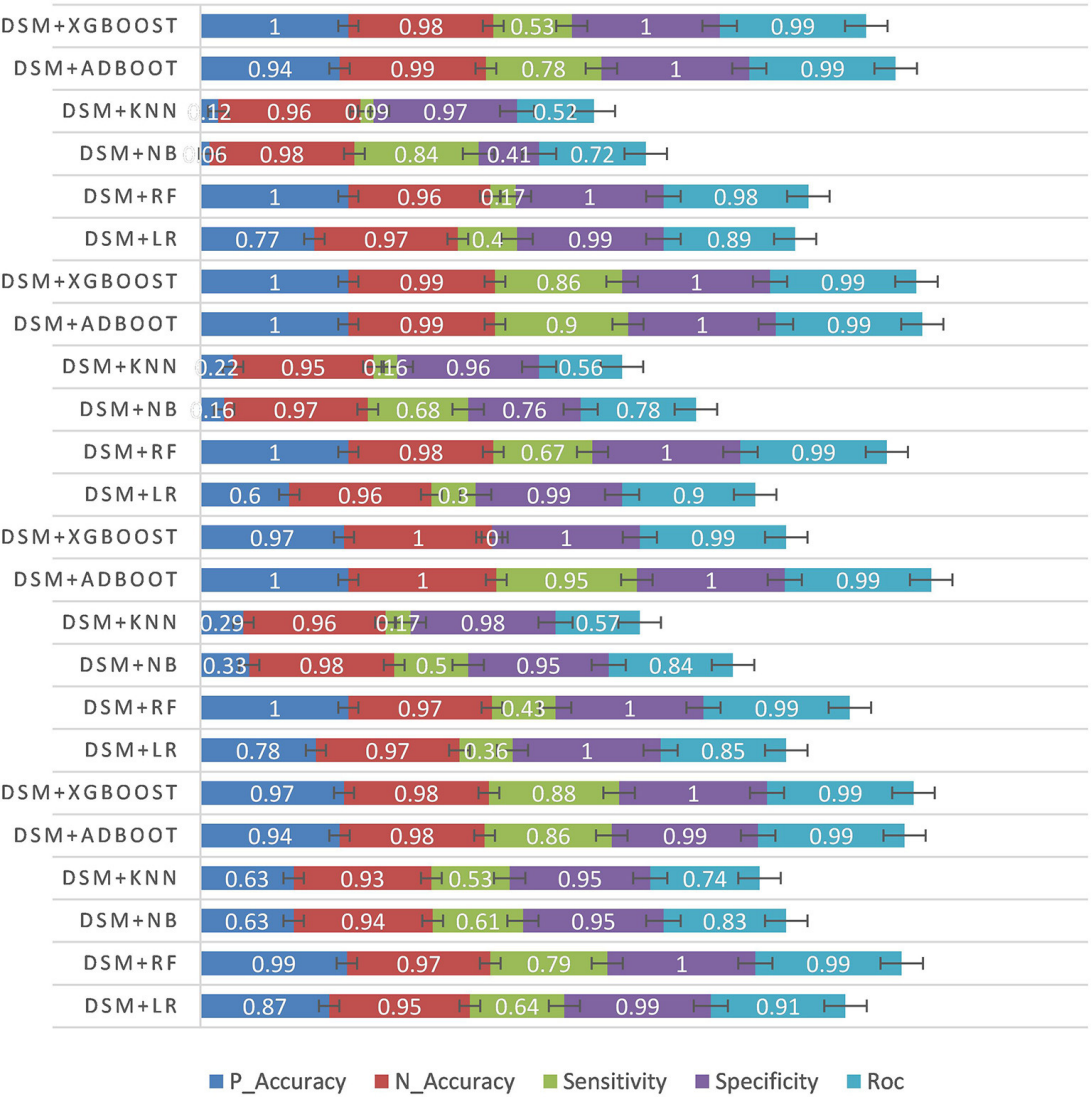
Temporal data experiment results.

In the second experiment, the data statistical model was used to conduct the experiment based on the patient's intraoperative temporal data. The experimental results are shown in Table 4 and Figure 5.

**Table 4:** Temporal data experiments results.

**Illness**	**Classifier**	**P_Accuracy**	**N_Accuracy**	**Sensitivity**	**Specificity**	**ROC**
Heart failure	DSM + LR	0.87	0.95	0.64	0.99	0.91
	DSM + RF	0.99	0.97	0.79	1	0.99
	DSM + NB	0.63	0.94	0.61	0.95	0.83
	DSM + KNN	0.63	0.93	0.53	0.95	0.74
	DSM + Adaboost	0.94	0.98	0.86	0.99	0.99
	DSM + XGBOOST	0.97	0.98	0.88	1	0.99
Liver failure	DSM + LR	0.78	0.97	0.36	1	0.85
	DSM + RF	1	0.97	0.43	1	0.99
	DSM + NB	0.33	0.98	0.5	0.95	0.84
	DSM + KNN	0.29	0.96	0.17	0.98	0.57
	DSM + Adaboost	1	1	0.95	1	0.99
	DSM + XGBOOST	0.97	1	0.9.	1	0.99
Renal failure	DSM + LR	0.6	0.96	0.3	0.99	0.9
	DSM + RF	1	0.98	0.67	1	0.99
	DSM + NB	0.16	0.97	0.68	0.76	0.78
	DSM + KNN	0.22	0.95	0.16	0.96	0.56
	DSM + Adaboost	1	0.99	0.9	1	0.99
	DSM + XGBOOST	1	0.99	0.86	1	0.99
Respiratory failure	DSM + LR	0.77	0.97	0.4	0.99	0.89
	DSM + RF	1	0.96	0.17	1	0.98
	DSM + NB	0.06	0.98	0.84	0.41	0.72
	DSM + KNN	0.12	0.96	0.09	0.97	0.52
	DSM + Adaboost	0.94	0.99	0.78	1	0.99
	DSM + XGBOOST	1	0.98	0.53	1	0.99

#### 3.2.3. Experiment 3: Unstructured data experiments and results

**Figure 6:**
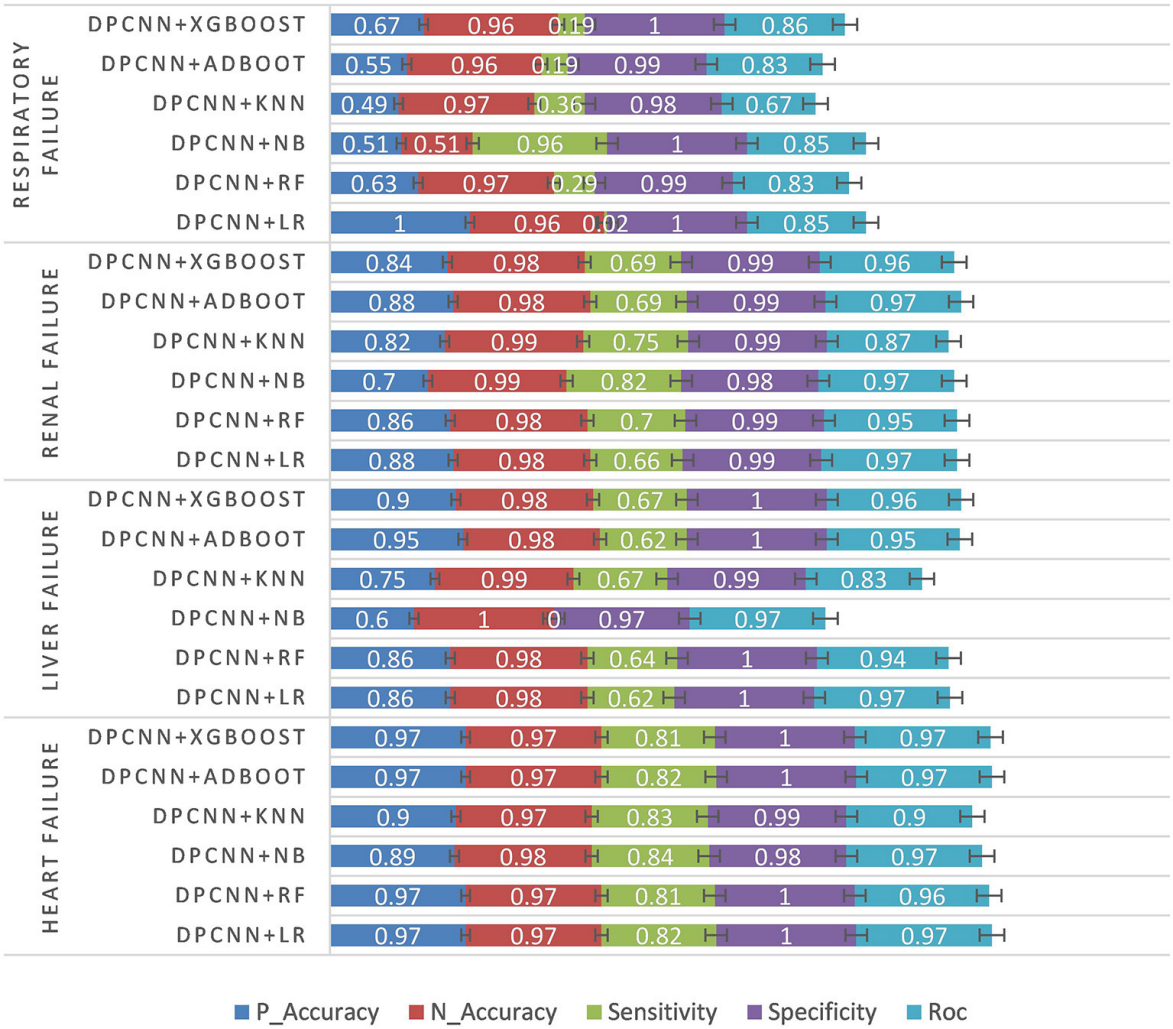
Unstructured data experiment results.

The third experiment was conducted using the unstructured text data of patients. First, text feature extraction was performed based on DPCNN, and then critical illness prediction was performed based on different classifiers. See Table 5 and Figure 6 for specific experimental results.

**Table 5:** Unstructured data experiments results.

**Illness**	**Classifier**	**P_Accuracy**	**N_Accuracy**	**Sensitivity**	**Specificity**	**ROC**
Heart failure	DPCNN + LR	0.97	0.97	0.82	1	0.97
	DPCNN + RF	0.97	0.97	0.81	1	0.96
	DPCNN + NB	0.89	0.98	0.84	0.98	0.97
	DPCNN + KNN	0.9	0.97	0.83	0.99	0.9
	DPCNN + Adaboost	0.97	0.97	0.82	1	0.97
	DPCNN + XGBOOST	0.97	0.97	0.81	1	0.97
Liver failure	DPCNN + LR	0.86	0.98	0.62	1	0.97
	DPCNN + RF	0.86	0.98	0.64	1	0.94
	DPCNN + NB	0.6	1	0.9.	0.97	0.97
	DPCNN + KNN	0.75	0.99	0.67	0.99	0.83
	DPCNN + Adaboost	0.95	0.98	0.62	1	0.95
	DPCNN + XGBOOST	0.9	0.98	0.67	1	0.96
Renal failure	DPCNN + LR	0.88	0.98	0.66	0.99	0.97
	DPCNN + RF	0.86	0.98	0.7	0.99	0.95
	DPCNN + NB	0.7	0.99	0.82	0.98	0.97
	DPCNN + KNN	0.82	0.99	0.75	0.99	0.87
	DPCNN + Adaboost	0.88	0.98	0.69	0.99	0.97
	DPCNN + XGBOOST	0.84	0.98	0.69	0.99	0.96
Respiratory failure	DPCNN + LR	1	0.96	0.02	1	0.85
	DPCNN + RF	0.63	0.97	0.29	0.99	0.83
	DPCNN + NB	0.51	0.51	0.96	1	0.85
	DPCNN + KNN	0.49	0.97	0.36	0.98	0.67
	DPCNN + Adaboost	0.55	0.96	0.19	0.99	0.83
	DPCNN + XGBOOST	0.67	0.96	0.19	1	0.86

#### 3.2.4. Experiment 4: Multimodal data experiments and results

**Figure 7:**
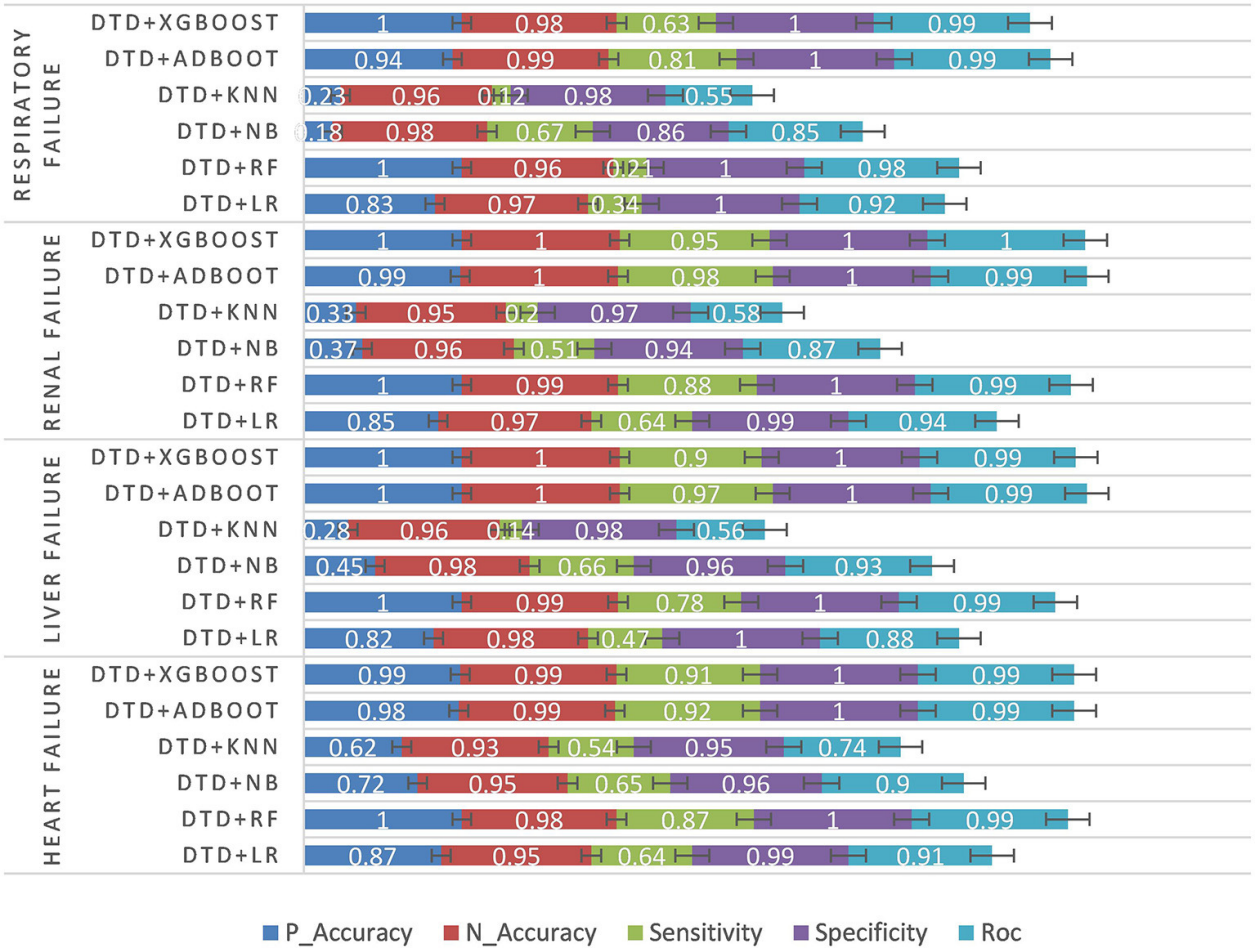
Multimodal data experiment results.

Finally, the experiment was carried out by fusing the characteristics of structured numerical type, unstructured text, and intraoperative monitoring data. DTD is the abbreviation of DPCNN + TBFS + DSM. See Table 6 and Figure 7 for the experimental results.

**Table 6:** Multimodal data experiments results.

**Illness**	**Classifier**	**P_Accuracy**	**N_Accuracy**	**Sensitivity**	**Specificity**	**ROC**
Heart failure	DTD + LR	0.87	0.95	0.64	0.99	0.91
	DTD + RF	1	0.98	0.87	1	0.99
	DTD + NB	0.72	0.95	0.65	0.96	0.9
	DTD + KNN	0.62	0.93	0.54	0.95	0.74
	DTD + Adaboost	0.98	0.99	0.92	1	0.99
	DTD + XGBOOST	0.99	0.99	0.91	1	0.99
Liver failure	DTD + LR	0.82	0.98	0.47	1	0.88
	DTD + RF	1	0.99	0.78	1	0.99
	DTD + NB	0.45	0.98	0.66	0.96	0.93
	DTD + KNN	0.28	0.96	0.14	0.98	0.56
	DTD + Adaboost	1	1	0.97	1	0.99
	DTD + XGBOOST	1	1	0.9	1	0.99
Renal failure	DTD + LR	0.85	0.97	0.64	0.99	0.94
	DTD + RF	1	0.99	0.88	1	0.99
	DTD + NB	0.37	0.96	0.51	0.94	0.87
	DTD + KNN	0.33	0.95	0.2	0.97	0.58
	DTD + Adaboost	0.99	1	0.98	1	0.99
	DTD + XGBOOST	1	1	0.95	1	1
Respiratory failure	DTD + LR	0.83	0.97	0.34	1	0.92
	DTD + RF	1	0.96	0.21	1	0.98
	DTD + NB	0.18	0.98	0.67	0.86	0.85
	DTD + KNN	0.23	0.96	0.12	0.98	0.55
	DTD + Adaboost	0.94	0.99	0.81	1	0.99
	DTD + XGBOOST	1	0.98	0.63	1	0.99

**Figure 8:**
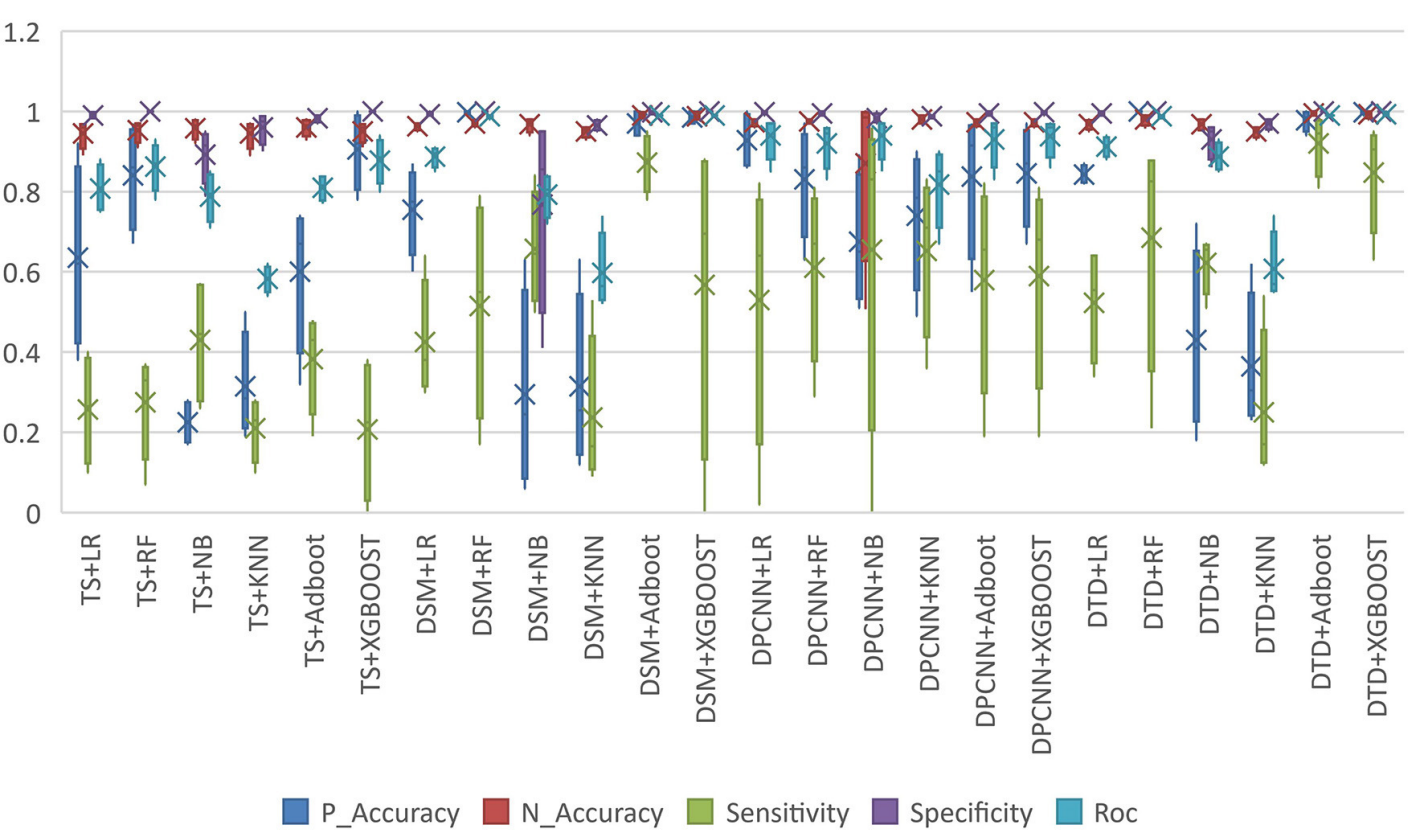
Model performance comparison.

According to the structured experimental results in Table 3, the sensitivity of all the models was lower, but the ROC value of the XGBOOST model was higher than that of the other models. Furthermore, due to a large number of missing values in the dataset, the LR model was filled with the value of −1 to process the missing values, which is worse than the tree model. Therefore, based on preoperative patient test data, the tree model is more suitable for surgical patients with a missing value. The experimental results from the intraoperative monitoring time series data in Table 4 show that the performance of each model was better than that of the preoperative prediction model, but the sensitivity was still low. As shown in Table 5, non-structural textual data based on preoperative examination can be used to predict postoperative heart failure. The preoperative text included preoperative diagnostic information and electrocardiogram examination conclusions for patients. Furthermore, both the electrocardiogram and the preoperative diagnosis of the positive patient contain information about the patient's heart disease, and the preoperative diagnosis and electrocardiogram examination conclusions of a negative patient are normal, so prediction of heart failure based on the text was very effective. In order to further integrate the patient diagnostic information, in this article, all diagnostic information of patients was integrated, as shown in Table 6 and Figure 8. It can be concluded that the prediction effect after fusion was better. The preoperative and intraoperative diagnostic data of patients can improve the prediction of critical illness.

## 4. Discussion and conclusion

The prediction of critical events in the perioperative period is a complex process. Whether serious adverse events occur during or after an operation depends entirely on the experience accumulation and judgment of doctors. The predictions of experienced doctors have high accuracy but the predictions of inexperienced doctors have low accuracy. Therefore, based on a machine learning method and multi-modal data of patients, this article built a prediction model for critical adverse events in patients so that the risk of critical events can be predicted for any patient directly based on the preoperative and intraoperative characteristic data. First, the preoperative patient data were preprocessed, and the patient data was divided into numerical structured data and text unstructured data. Then, through the fusion of text features extracted by a deep neural network and pure numerical type features extracted by feature engineering, the risk prediction model was trained by the method of supervised learning, and analyzed whether the perioperative patients are at risk of critical illness and give an early warning. The proposed model in this article was based on the data of critically ill patients in a Class A tertiary hospital and it is not suitable for direct promotion and application without multi-center data verification. However, the model can be learned and extended according to different data sets of critical events. There are also the following directions for further research in future work. In different stages of the perioperative period, different critical diseases have different effects on patients. At present, this work only classifies and predicts the occurrence of critical illness during or after an operation based on the preoperative examination data of patients, but does not discuss the specific time when the patient was at risk for critical illness. This is the direction of our future work.

## Data availability statement

The original contributions presented in the study are included in the article/supplementary material, further inquiries can be directed to the corresponding author.

## Ethics statement

Written informed consent was obtained from the individual(s) for the publication of any potentially identifiable images or data included in this article.

## Author contributions

Study concept, design, and funding: Y-wC and BY. Analysis, interpretation of data, and technical support: Y-wC and L-qX. Writing original manuscript: Y-wC. Revision of manuscript: BY. All authors contributed to the article and approved the submitted version.
